# Supplementary material for: On the power of multipartite entanglement for pseudotelepathy
Source: Proc Natl Acad Sci U S A. 2026 Sep 24;123(39):e2607361123. doi: 10.1073/pnas.2607361123 (PMC13624733; doi:10.1073/pnas.2607361123)
Supplement: Supplementary file 1 [file pnas.2607361123.sapp.pdf]

PNAS

Supporting Information for

On the power of multipartite entanglement for pseudotelepathy

Gilles Brassard, Xavier Coiteux-Roy and Rémi Ligez

Gilles Brassard.
E-mail: brassard@iro.umontreal.ca

This PDF file includes:

Supporting text
SI References

Supporting Information Text

1. The GHZ–Mermin game cannot be won with arbitrary bipartite quantum resources.

A significant property of the GHZ–Mermin game is its *rigidity*: the winning quantum strategy, and in particular the quantum state it uses, are essentially unique. This notion was introduced by Mayers and Yao under the name of *self testing* (1), where they proved the rigidity of the optimal quantum strategy to win the CHSH game with probability $\cos^2(\pi/8)$. More to the point, as shown by Colbeck and Kent (2), if Alice, Bob and Charlie win the GHZ–Mermin game with the shared state $|\psi\rangle$ on $\mathcal{H}_A \otimes \mathcal{H}_B \otimes \mathcal{H}_C$, then there exist local isometries

$$\Phi_A : \mathcal{H}_A \to \mathbb{C}^2 \otimes \mathcal{H}'_A \,, \Phi_B : \mathcal{H}_B \to \mathbb{C}^2 \otimes \mathcal{H}'_B \,, \Phi_C : \mathcal{H}_C \to \mathbb{C}^2 \otimes \mathcal{H}'_C \,,$$

and some state $|\text{junk}\rangle$ on $\mathcal{H}'_A \otimes \mathcal{H}'_B \otimes \mathcal{H}'_C$ such that

$$(\Phi_A \otimes \Phi_B \otimes \Phi_C)\,|\psi\rangle = |\text{GHZ}\rangle \otimes |\text{junk}\rangle \;.$$

Furthermore, the GHZ state cannot be generated amongst three non-communicating participants with only bipartite quantum resources (3). Combining these two facts, we conclude that the GHZ–Mermin game cannot be won using arbitrary bipartite quantum resources.

2. The majority game.

Recall that the majority game is a 3-player nonlocal game in which Alice, Bob and Charlie each receive an input bit $x, y, z \in \{0,1\}$. Each player must output a bit $a, b, c \in \{0,1\}$ such that $a \oplus b \oplus c = \text{Maj}(x,y,z)$, where $\text{Maj}(x,y,z) = 1$ if at least two of x, y, z are equal to 1, and $\text{Maj}(x,y,z) = 0$ otherwise.

Theorem 2.1. *No quantum strategy can win the majority game with probability greater than* $\cos^2(\pi/8)$.

Proof. For the two notions of winning probability considered here, any strategy for the majority game that wins with probability p can be transformed into one that wins the CHSH game with probability at least p, which is not possible for $p > \cos^2(\pi/8)$, according to Tsirelson's bound (4). We now describe these (black-box) reductions explicitly, first for the worst-case winning probability and then for the average-case winning probability under uniform input distribution.

Recall that the CHSH game is a 2-player game in which Alice′ and Bob′ each receive an input bit $x', y' \in \{0,1\}$ and must output a bit $a', b' \in \{0,1\}$ satisfying $a' \oplus b' = x' \wedge y'$.

Assume Alice, Bob and Charlie win the majority game with worst-case probability p. That is, they have access to three boxes that, on inputs x, y and z, respectively, output a, b and c, respectively, satisfying

$$\min_{x,y,z} \Pr[a \oplus b \oplus c = \text{Maj}(x,y,z)] = p\,.$$

In an instance of the CHSH game, Alice′ receives x' and Bob′ receives y'. Alice′ inputs $x = x'$ and $z = 0$ in Alice's and Charlie's majority game's boxes, whereas Bob′ inputs $y = y'$ in Bob's. Alice′ obtains a and c from Alice's and Charlie's boxes, and outputs $a' = a \oplus c$. Bob′ obtains b from Bob's box, and outputs $b' = b$. The probability that they win their CHSH instance is given by

$$\begin{aligned}\Pr\left[a' \oplus b' = x' \wedge y'\right] &= \Pr[(a \oplus c) \oplus b = x \wedge y] \\ &= \Pr[a \oplus b \oplus c = x \wedge y] \\ &= \Pr[a \oplus b \oplus c = \text{Maj}(x,y,0)] \geq p\,.\end{aligned}$$

Hence, they win the CHSH game with probability at least p.

In the case that the winning probability is taken over the uniform distribution on the inputs, Alice, Bob and Charlie's boxes for the majority game verify

$$\sum_{x,y,z} \frac{1}{8} \Pr[a \oplus b \oplus c = \text{Maj}(x,y,z)] = p\,,$$

which can be rewritten as

$$\frac{1}{2}\sum_{x,y} \frac{1}{4} \Pr[a \oplus b \oplus c = \text{Maj}(x,y,0)] + \frac{1}{2}\sum_{x,y} \frac{1}{4} \Pr[a \oplus b \oplus c = \text{Maj}(x,y,1)] = p\,.$$

Alice′ and Bob′ supplement their majority game boxes with shared randomness. Since the shared randomness can be simulated by measuring a shared quantum state, this does not grant the reduction any additional power. Concretely, they sample a shared random variable $\Lambda = \{0,1\}$ with $\Pr[\Lambda = 0] = \Pr[\Lambda = 1] = 1/2$. On one hand, when $\Lambda = 0$, Alice′ inputs $x = x'$ and $z = 0$ in Alice's and Charlie's majority game's boxes, whereas Bob′ inputs $y = y'$ in Bob's. Alice′ obtains a and c from Alice's and Charlie's boxes and outputs $a' = a \oplus c$. Bob′ obtains b from Bob's box, and outputs $b' = b$. On the other hand, when $\Lambda = 1$, Alice′ inputs $x = x' \oplus 1$ and $z = 1$ in Alice's and Charlie's majority game's boxes, whereas Bob′ inputs $y = y' \oplus 1$ in

Bob's. Alice′ obtains a and c from Alice and Charlie's boxes and outputs $a' = a \oplus c \oplus 1$. Bob′ obtains b from Bob's box and outputs $b' = b$. The probability that they win the CHSH game is given by

$$
\begin{aligned}
&\sum_{x',y'} \frac{1}{4} \Pr\left[a' \oplus b' = x' \wedge y'\right] \\
&= \frac{1}{2} \sum_{x',y'} \frac{1}{4} \Pr\left[a' \oplus b' = x' \wedge y' \mid \Lambda = 0\right] + \frac{1}{2} \sum_{x',y'} \frac{1}{4} \Pr\left[a' \oplus b' = x' \wedge y' \mid \Lambda = 1\right] \\
&= \frac{1}{2} \sum_{x,y} \frac{1}{4} \Pr[(a \oplus c) \oplus b = x \wedge y \mid \Lambda = 0] + \frac{1}{2} \sum_{x,y} \frac{1}{4} \Pr[(a \oplus c \oplus 1) \oplus b = (x \oplus 1) \wedge (y \oplus 1) \mid \Lambda = 1] \\
&= \frac{1}{2} \sum_{x,y} \frac{1}{4} \Pr[a \oplus b \oplus c = x \wedge y \mid \Lambda = 0] + \frac{1}{2} \sum_{x,y} \frac{1}{4} \Pr[a \oplus b \oplus c = x \vee y \mid \Lambda = 1] \\
&= \frac{1}{2} \sum_{x,y} \frac{1}{4} \Pr[a \oplus b \oplus c = \mathrm{Maj}(x,y,0)] + \frac{1}{2} \sum_{x,y} \frac{1}{4} \Pr[a \oplus b \oplus c = \mathrm{Maj}(x,y,1)] \\
&= p\,.
\end{aligned}
$$

Hence, they win the CHSH game with probability p, concluding the reduction. □

Theorem 2.2. *A quantum strategy can win the majority game with probability* $\cos^2(\pi/8)$.

Proof. The quantum strategy is the following. Alice, Bob and Charlie share the quantum state

$$\tfrac{1}{\sqrt{2}} |000\rangle + \tfrac{1-i}{2} |111\rangle \,.$$

Each player, on input 1, applies a $\sqrt{Z}$ gate (which sends $|0\rangle$ to $|0\rangle$ and $|1\rangle$ to $i\,|1\rangle$). Afterwards, regardless of the input, each player applies a Hadamard gate and measures in the computational basis. The measurement outcome is then the player's output. A tedious, yet straightforward computation shows that this strategy wins with probability $\cos^2(\pi/8)$ for each possible input. □

Theorem 2.3. *A strategy using only three PR–boxes can win the majority game.*

Proof. The strategy is the following. Each pair of players shares a PR–box. Each player inputs his input bit into both of his boxes and outputs the XOR of the two outputs from his boxes. Let $x, y, z \in \{0,1\}$ be the inputs of Alice, Bob and Charlie, respectively. Let the outputs of Alice's boxes be a_1, a_2, Bob's be b_1, b_2 and Charlie's be c_1, c_2. By the PR–box correlations,

$$
\begin{aligned}
a_1 \oplus b_1 &= x \wedge y\,, \\
a_2 \oplus c_1 &= x \wedge z\,, \\
b_2 \oplus c_2 &= y \wedge z\,.
\end{aligned}
$$

Thus,

$$
\begin{aligned}
(a_1 \oplus a_2) \oplus (b_1 \oplus b_2) \oplus (c_1 \oplus c_2) &= (a_1 \oplus b_1) \oplus (a_2 \oplus c_1) \oplus (b_2 \oplus c_2) \\
&= (x \wedge y) \oplus (x \wedge z) \oplus (y \wedge z) \\
&= \mathrm{Maj}(x,y,z)\,.
\end{aligned}
$$

Hence, the players win the majority game. □

3. The combination of the magic square game with the equality game can be won using a single PR–box.

Inspired by the construction of Ref. (5), we define a nonlocal game that combines the magic square game and the equality game. The game involves three players: Alice, Bob and Charlie. Alice receives a row $x \in \{1,2,3\}$ in the magic square, while Bob receives a column $y \in \{1,2,3\}$. In addition, Bob and Charlie receive a common input bit z, which indicates whether Alice and Bob are to play the magic square game (when $z = 0$) or they are all to play two parallel instances of the equality game (when $z = 1$). The promise is that $x = 1$ whenever $z = 1$. Each player must output two bits (a_1, a_2 for Alice, b_1, b_2 for Bob, and c_1, c_2 for Charlie) such that if $z = 0$, we ignore Charlie's output and ask that Alice and Bob win their magic square game instance (the third bit is determined by the required parity, so it suffices to output two bits each) and if $z = 1$, they all have to play the equality game twice (i.e. their outputs must satisfy $a_1 = b_1 = c_1$ and $a_2 = b_2 = c_2$).

Although the game as described above does not admit a quantum winning strategy, a slight modification suffices to make the game quantumly winnable. This modification allows us to obtain a pseudotelepathic game, since the modified game still admits no classical winning strategy. Bob is given two additional input bits y_1 and y_2, and the rule of the game is modified so that the players win the game whenever $z = 0$ and $(y_1, y_2) \neq (c_1, c_2)$. Two GHZ states, each shared between Alice, Bob and Charlie, are sufficient to win this modified game. When $z = 1$, Bob and Charlie measure their respective qubits in the

computational basis and output their measurement outcomes. When $z = 0$, Charlie measures his qubits in the Hadamard basis and outputs the resulting bits. In this case, Bob may treat his input bits y_1, y_2 as Charlie's outputs since the players win whenever these differ. Bob can therefore apply an appropriate local transformation to his qubits so that he and Alice share two EPR states. Alice and Bob can then win their magic square game instance with a strategy that uses two EPR states and in which Alice measures her qubits in the computational basis when given $x = 1$. This choice of strategy ensures that the players also win the game when $z = 1$ since Alice receives $x = 1$ whenever $z = 1$, even though she does not know which game they are playing. Such a strategy for the magic square game is well known (6).

We now describe a strategy that wins this game using a single PR–box shared between Alice and Bob. Intuitively, this is possible because the magic square game with the first row fixed can be won with a single PR–box. This fact allows Alice to answer fixed outputs when $x = 1$, while Bob and Charlie can answer the same fixed outputs when $z = 1$, and still enables Alice and Bob to win the magic square game when $x \neq 1$. Concretely, we fix the first row of the magic square to be $(0, 0, 0)$ and the first column to be $(0, 0, 1)$, the remaining constraints are then equivalent to those of the CHSH game. Formally, Alice outputs $(0, 0)$ when $x = 1$. When $x = 2$, she inputs 0 into her PR–box to obtain bit a_2 and outputs $(0, a_2)$. When $x = 3$, she inputs 1 in her PR–box to obtain bit a_2 and outputs $(1, a_2)$. Bob, ignoring his auxiliary inputs y_1 and y_2, outputs $(0, 0)$ when $z = 1$ or $y = 1$. Otherwise, he inputs $3 - y$ into his PR–box to obtain bit b_2 and outputs $(0, b_2)$. Charlie outputs $(0, 0)$ regardless of his inputs. It is immediate that the players win the game whenever $z = 1$, since in that case $x = 1$ and all three players output $(0, 0)$. A simple calculation also shows that Alice and Bob win the magic square game whenever $z = 0$. Hence, this combination of the magic square game with the equality game can be won using a single PR–box, even though it is pseudotelepathic. Therefore, it does not constitute a suitable candidate for exhibiting a separation between tripartite entanglement and arbitrary nonsignalling bipartite correlations.

4. Lemmas on nonlocal games.

We begin by introducing notation used throughout this section. For a general N-player nonlocal game, let X_i and A_i denote the input and output sets of the i-th player, respectively. The probability that the players output $(a_1, \ldots, a_N) \in A_1 \times \ldots \times A_N$ on input $(x_1, \ldots, x_N) \in X_1 \times \ldots \times X_N$ is denoted by

$$\Pr_{A_1 \ldots A_N | X_1 \ldots X_N}(a_1, \ldots, a_N \mid x_1, \ldots, x_N) \,.$$

Similarly, when the players have access to shared randomness $\lambda \in \Lambda$, sampled before the game instance begins, we write instead

$$\Pr_{A_1 \ldots A_N | X_1 \ldots X_N \Lambda}(a_1, \ldots, a_N \mid x_1, \ldots, x_N, \lambda) \,.$$

For any subset of players $S \subseteq \{1, \ldots, N\}$, we use the natural shorthand $\Pr_{A_S|X_S}$ to denote the corresponding marginal distribution over the inputs and outputs of the players in S. For example, $\Pr_{A_i A_j | X_i X_j}(a_i, a_j \mid x_i, x_j)$ denotes the probability of players i and j outputting a_i, a_j on inputs x_i, x_j.

Lemma 4.1. *If there exists a winning strategy for a nonlocal game, then there also exists one that uses no shared randomness, but otherwise all the same resources.*

Proof. This follows easily from the fact that we can write

$$\Pr_{A_1 \ldots A_N | X_1 \ldots X_N}(a_1, \ldots, a_N \mid x_1, \ldots, x_N) = \sum_{\lambda \in \Lambda} \Pr_\Lambda(\lambda) \cdot \Pr_{A_1 \ldots A_N | X_1 \ldots X_N \Lambda}(a_1, \ldots, a_N \mid x_1, \ldots, x_N, \lambda) \,.$$

If the strategy is winning, then for every λ such that $\Pr_\Lambda(\lambda) > 0$, any output $(a_1, \ldots, a_N)$ with

$$\Pr_{A_1 \ldots A_N | X_1 \ldots X_N \Lambda}(a_1, \ldots, a_N \mid x_1, \ldots, x_N, \lambda) > 0$$

must be accepted for the input $(x_1, \ldots, x_N)$. Thus, fixing any such λ yields a strategy without shared randomness that wins the game. □

Lemma 4.2. *If there exists a nonsignalling strategy for a game in which each player has only two possible inputs and behaves deterministically on one of them, then there also exists a classical strategy that reproduces exactly the same output distribution for any given input.*

Proof. Consider an N-player nonlocal game in which each player has exactly two inputs. Assume that there exists a strategy for this game such that each player behaves deterministically on one of them. We refer to this strategy as the *initial* strategy. Our goal is to construct a fully classical strategy that reproduces exactly the same output distribution for every input. Since each player has two possible inputs, we may, without loss of generality, label them by $X_i = \{0, 1\}$, for all $1 \leq i \leq N$. Moreover, for each player i, we assume without loss of generality that the initial strategy is deterministic on input $x_i = 1$. Given inputs $x_1, \ldots, x_N$, we denote by $j_1, \ldots, j_m$ the players who received input 0 and by $k_1, \ldots, k_n$ those who received input 1, with $m + n = N$. For notational convenience, we shall write $A_{j_1}^{j_m}$ for $A_{j_1} \ldots A_{j_m}$, $A_{k_1}^{k_n}$ for $A_{k_1} \ldots A_{k_n}$, $X_{j_1}^{j_m}$ for $X_{j_1} \ldots X_{j_m}$ and $X_{k_1}^{k_n}$ for $X_{k_1} \ldots X_{k_n}$. Since each player i is deterministic on input $x_i = 1$, there exists an output $\hat{a}_i \in A_i$ such that

$$\Pr_{A_i|X_i}(a_i \mid 1) = \delta(a_i, \hat{a}_i) \,,$$

where δ denotes the Kronecker delta. We begin by establishing a fact about the initial strategy. For any input $(x_1,\dots,x_N)\in X_1\times\ldots\times X_N$ and output $(a_1,\dots,a_N)\in A_1\times\ldots\times A_N$,

$$
\begin{aligned}
&\Pr_{A_1\ldots A_N|X_1\ldots X_N}(a_1,\dots,a_N \mid x_1,\dots,x_N) && [1]\\
&= \Pr_{A_{j_1}\ldots A_{j_m}A_{k_1}\ldots A_{k_n}|X_{j_1}\ldots X_{j_m}X_{k_1}\ldots X_{k_n}}(a_{j_1},\dots,a_{j_m},a_{k_1},\dots,a_{k_n} \mid 0,\dots,0,1,\dots,1) && [2]\\
&= \Pr_{A_{j_1}^{j_m}|X_{j_1}^{j_m}X_{k_1}^{k_n}}(a_{j_1},\dots,a_{j_m} \mid 0,\dots,0,1,\dots,1)\cdot \Pr_{A_{k_1}^{k_n}|A_{j_1}^{j_m}X_{j_1}^{j_m}X_{k_1}^{k_n}}(a_{k_1},\dots,a_{k_n} \mid a_{j_1},\dots,a_{j_m},0,\dots,0,1,\dots,1) && [3]\\
&= \Pr_{A_{j_1}^{j_m}|X_{j_1}^{j_m}}(a_{j_1},\dots,a_{j_m} \mid 0,\dots,0)\cdot \Pr_{A_{k_1}^{k_n}|A_{j_1}^{j_m}X_{j_1}^{j_m}X_{k_1}^{k_n}}(a_{k_1},\dots,a_{k_n} \mid a_{j_1},\dots,a_{j_m},0,\dots,0,1,\dots,1) && [4]\\
&= \Pr_{A_{j_1}^{j_m}|X_{j_1}^{j_m}}(a_{j_1},\dots,a_{j_m} \mid 0,\dots,0)\cdot \prod_{\ell=1}^{n}\delta(a_{k_\ell},\hat{a}_{k_\ell})\,, && [5]
\end{aligned}
$$

where Eq. (2) is obtained by a simple reordering of the players; Eq. (3) follows from an application of Bayes' theorem; Eq. (4) follows from the nonsignalling condition applied to the left-hand term; and Eq. (5) is a consequence of the fact that the i-th player systematically outputs $\hat{a}_i$ on input 1. We shall make use of this equation later on in the proof.

We now define our new classical strategy, which satisfies the lemma's statement. The strategy is denoted by $\widehat{\Pr}$, and will make use of shared randomness Λ, which is defined on $A_1\times\ldots\times A_N$. We write $\lambda\in\Lambda$ as $\lambda=(\lambda_1,\dots,\lambda_N)$ with $\lambda_i\in A_i$ for each i. The probability distribution on Λ and the players' strategy are defined as follows:

$$
\begin{aligned}
\widehat{\Pr}_\Lambda(\lambda_1,\dots,\lambda_N) &:= \Pr_{A_1\ldots A_N|X_1\ldots X_N}(\lambda_1,\dots,\lambda_N \mid 0,\dots,0)\,,\\
\widehat{\Pr}_{A_i|X_i\Lambda}(a_i \mid 0,\lambda_1,\dots\lambda_N) &:= \delta(a_i,\lambda_i)\,,\\
\widehat{\Pr}_{A_i|X_i\Lambda}(a_i \mid 1,\lambda_1,\dots\lambda_N) &:= \delta(a_i,\hat{a}_i)\,.
\end{aligned}
$$

That is, in the new classical strategy, the shared randomness is distributed as the players' joint output when they all receive input 0 in the initial strategy. Player i outputs the i-th component of the shared randomness, λ_i, if $x_i=0$, and outputs $\hat{a}_i$ if $x_i=1$. What remains to be shown is that this strategy reproduces exactly the same output distribution for any given input as the initial strategy. We have

$$
\begin{aligned}
&\widehat{\Pr}_{A_1\ldots A_N|X_1\ldots X_N}(a_1,\dots,a_N \mid x_1,\dots,x_N) && [6]\\
&= \sum_{\lambda_1,\dots,\lambda_N}\widehat{\Pr}_\Lambda(\lambda_1,\dots,\lambda_N)\cdot\widehat{\Pr}_{A_1\ldots A_N|X_1\ldots X_N\Lambda}(a_1,\dots,a_N \mid x_1,\dots,x_N,\lambda_1,\dots,\lambda_N) && [7]\\
&= \sum_{\lambda_1,\dots,\lambda_N}\Pr_{A_1\ldots A_N|X_1\ldots X_N}(\lambda_1,\dots,\lambda_N \mid 0,\dots,0)\cdot\prod_{t=1}^{m}\delta(a_{j_t},\lambda_{j_t})\cdot\prod_{\ell=1}^{n}\delta(a_{k_\ell},\hat{a}_{k_\ell}) && [8]\\
&= \sum_{\lambda_{k_1},\dots,\lambda_{k_n}}\Pr_{A_{j_1}^{j_m}A_{k_1}^{k_n}|X_{j_1}^{j_m}X_{k_1}^{k_n}}(a_{j_1},\dots,a_{j_m},\lambda_{k_1},\dots,\lambda_{k_n} \mid 0,\dots,0,0,\dots,0)\cdot\prod_{\ell=1}^{n}\delta(a_{k_\ell},\hat{a}_{k_\ell}) && [9]\\
&= \Pr_{A_{j_1}^{j_m}|X_{j_1}^{j_m}X_{k_1}^{k_n}}(a_{j_1},\dots,a_{j_m} \mid 0,\dots,0,0,\dots,0)\cdot\prod_{\ell=1}^{n}\delta(a_{k_\ell},\hat{a}_{k_\ell}) && [10]\\
&= \Pr_{A_{j_1}^{j_m}|X_{j_1}^{j_m}}(a_{j_1},\dots,a_{j_m} \mid 0,\dots,0)\cdot\prod_{\ell=1}^{n}\delta(a_{k_\ell},\hat{a}_{k_\ell}) && [11]\\
&= \Pr_{A_1\ldots A_N|X_1\ldots X_N}(a_1,\dots,a_N \mid x_1,\dots,x_N)\,, && [12]
\end{aligned}
$$

where Eq. (7) follows directly from the definition of shared randomness; Eq. (8) uses the definition of the new strategy $\widehat{\Pr}$, where, by construction, player i outputs λ_i on input 0 and $\hat{a}_i$ on input 1; Eq. (9) follows from the observation that only terms satisfying $\lambda_{j_t}=a_{j_t}$ for $t\in\{1,\dots,m\}$ have nonzero contribution; Eq. (10) is obtained by summing over all possible outputs of players $k_1,\dots,k_n$; Eq. (11) follows from the nonsignalling condition applied to players $j_1,\dots,j_m$; and finally, Eq. (12) is an immediate consequence of Eq. (5) being equal to Eq. (1).

Therefore, the new classical strategy reproduces exactly the output distribution of the initial strategy, which concludes the proof. □

Lemma 4.3. *If there exists a nonsignalling strategy for a game in which each player has at most one input on which he does not behave deterministically, then there also exists a classical strategy that reproduces exactly the same output distribution for any given input.*

Proof. Following the lines of the proof of Lemma 4.2, consider an N-player nonlocal game. Assume that there exists a nonsignalling strategy for this game such that each player has at most one input on which he does not behave deterministically. We refer to this strategy as the *initial* strategy. Our goal is to construct a fully classical strategy that reproduces exactly the same output distribution for every input.

If all players are deterministic on all inputs, the conclusion is immediate. Otherwise, without loss of generality, the input sets are $X_i = \{0, 1, \ldots, d_i\}$ for $1 \le i \le N$. Moreover, also without loss of generality, we relabel the players and the inputs so that there exists $D \in \{1, \ldots, N\}$ such that players $1, \ldots, D$ are non-deterministic on input 0 and are deterministic on inputs different from 0, while players $D+1, \ldots, N$ are deterministic on all their inputs. Given inputs $x_1, \ldots, x_D$ for players $1, \ldots, D$, we denote by $j_1, \ldots, j_m$ the players who received input 0 and by $k_1, \ldots, k_n$ those who received an input different from 0, with $m + n = D$. For notational convenience, we shall write $A_{j_1}^{j_m}$ for $A_{j_1} \ldots A_{j_m}$, $A_{k_1}^{k_n}$ for $A_{k_1} \ldots A_{k_n}$, A_{D+1}^{N} for $A_{D+1} \ldots A_N$, $X_{j_1}^{j_m}$ for $X_{j_1} \ldots X_{j_m}$, $X_{k_1}^{k_n}$ for $X_{k_1} \ldots X_{k_n}$ and X_{D+1}^{N} for $X_{D+1} \ldots X_N$. By definition, when player i is deterministic on input x_i, there exists an output $\hat{a}_i^{(x_i)} \in A_i$ such that

$$\Pr_{A_i|X_i}(a_i \mid x_i) = \delta(a_i, \hat{a}_i^{(x_i)}) \,,$$

where δ denotes the Kronecker delta. We begin by establishing a fact about the initial strategy. For any input $(x_1, \ldots, x_N) \in X_1 \times \ldots \times X_N$ and output $(a_1, \ldots, a_N) \in A_1 \times \ldots \times A_N$,

$$\Pr_{A_1 \ldots A_N | X_1 \ldots X_N}(a_1, \ldots, a_N \mid x_1, \ldots, x_N) \tag{13}$$
$$= \Pr_{A_{j_1}^{j_m} A_{k_1}^{k_n} A_{D+1}^{N} | X_{j_1}^{j_m} X_{k_1}^{k_n} X_{D+1}^{N}}(a_{j_1}, \ldots, a_{j_m}, a_{k_1}, \ldots, a_{k_n}, a_{D+1}, \ldots, a_N \mid 0, \ldots, 0, x_{k_1}, \ldots, x_{k_n}, x_{D+1}, \ldots, x_N) \tag{14}$$
$$= \Pr_{A_{j_1}^{j_m} | X_{j_1}^{j_m} X_{k_1}^{k_n} X_{D+1}^{N}}(a_{j_1}, \ldots, a_{j_m} \mid 0, \ldots, 0, x_{k_1}, \ldots, x_{k_n}, x_{D+1}, \ldots, x_N) \cdot \Pr_{A_{k_1}^{k_n} A_{D+1}^{N} | A_{j_1}^{j_m} X_{j_1}^{j_m} X_{k_1}^{k_n} X_{D+1}^{N}}(a_{k_1}, \ldots, a_{k_n}, a_{D+1}, \ldots, a_N \mid a_{j_1}, \ldots, a_{j_m}, 0, \ldots, 0, x_{k_1}, \ldots, x_{k_n}, x_{D+1}, \ldots, x_N) \tag{15}$$
$$= \Pr_{A_{j_1}^{j_m} | X_{j_1}^{j_m}}(a_{j_1}, \ldots, a_{j_m} \mid 0, \ldots, 0) \cdot \Pr_{A_{k_1}^{k_n} A_{D+1}^{N} | A_{j_1}^{j_m} X_{j_1}^{j_m} X_{k_1}^{k_n} X_{D+1}^{N}}(a_{k_1}, \ldots, a_{k_n}, a_{D+1}, \ldots, a_N \mid a_{j_1}, \ldots, a_{j_m}, 0, \ldots, 0, x_{k_1}, \ldots, x_{k_n}, x_{D+1}, \ldots, x_N) \tag{16}$$
$$= \Pr_{A_{j_1}^{j_m} | X_{j_1}^{j_m}}(a_{j_1}, \ldots, a_{j_m} \mid 0, \ldots, 0) \cdot \prod_{\ell=1}^{n} \delta(a_{k_\ell}, \hat{a}_{k_\ell}^{(x_{k_\ell})}) \cdot \prod_{r=D+1}^{N} \delta(a_r, \hat{a}_r^{(x_r)}) \,, \tag{17}$$

where Eq. (14) is obtained by a simple reordering of the players; Eq. (15) follows from an application of Bayes' theorem; Eq. (16) follows from the nonsignalling condition applied to the left-hand term; and Eq. (17) is a consequence of the fact that players $k_1, \ldots, k_n$ and $D+1, \ldots, N$ are deterministic on their given input. We shall make use of this equation later on in the proof.

We now define our new classical strategy, which satisfies the lemma's statement. The strategy is denoted by $\widehat{\Pr}$, and will make use of shared randomness Λ, which is defined on $A_1 \times \ldots \times A_D$. We write $\lambda \in \Lambda$ as $\lambda = (\lambda_1, \ldots, \lambda_D)$ with $\lambda_i \in A_i$ for each $i \in \{1, \ldots, D\}$. The probability distribution on Λ and the players' strategy are defined as follows:

$$\begin{aligned}
\widehat{\Pr}_{\Lambda}(\lambda_1, \ldots, \lambda_D) &:= \Pr_{A_1 \ldots A_D | X_1 \ldots X_D}(\lambda_1, \ldots, \lambda_D \mid 0, \ldots, 0) \,, \\
\widehat{\Pr}_{A_i | X_i \Lambda}(a_i \mid 0, \lambda_1, \ldots \lambda_D) &:= \delta(a_i, \lambda_i) \text{ for } i \in \{1, \ldots, D\} \,, \\
\widehat{\Pr}_{A_i | X_i \Lambda}(a_i \mid x_i, \lambda_1, \ldots \lambda_D) &:= \delta(a_i, \hat{a}_i^{(x_i)}) \text{ for } i \in \{1, \ldots, D\} \text{ and } x_i \neq 0 \,, \\
\widehat{\Pr}_{A_i | X_i \Lambda}(a_i \mid x_i, \lambda_1, \ldots \lambda_D) &:= \delta(a_i, \hat{a}_i^{(x_i)}) \text{ for } i \in \{D+1, \ldots, N\} \,.
\end{aligned}$$

That is, in the new classical strategy, the shared randomness is distributed as the joint output of players $1, \ldots, D$ when they all receive input 0 in the initial strategy. For $i \in \{1, \ldots, D\}$, player i outputs the i-th component of the shared randomness, λ_i, if $x_i = 0$, and outputs $\hat{a}_i^{(x_i)}$ if $x_i \neq 0$. For $i \in \{D+1, \ldots, N\}$, player i outputs $\hat{a}_i^{(x_i)}$ on input x_i. What remains to be shown is that this strategy reproduces exactly the same output distribution for any given input as the initial strategy. We have

$$\widehat{\Pr}_{A_1 \ldots A_N | X_1 \ldots X_N}(a_1, \ldots, a_N \mid x_1, \ldots, x_N) \tag{18}$$
$$= \sum_{\lambda_1, \ldots, \lambda_D} \widehat{\Pr}_{\Lambda}(\lambda_1, \ldots, \lambda_D) \cdot \widehat{\Pr}_{A_1 \ldots A_N | X_1 \ldots X_N \Lambda}(a_1, \ldots, a_N \mid x_1, \ldots, x_N, \lambda_1, \ldots, \lambda_D) \tag{19}$$
$$= \sum_{\lambda_1, \ldots, \lambda_D} \Pr_{A_1 \ldots A_D | X_1 \ldots X_D}(\lambda_1, \ldots, \lambda_D \mid 0, \ldots, 0) \cdot \prod_{t=1}^{m} \delta(a_{j_t}, \lambda_{j_t}) \cdot \prod_{\ell=1}^{n} \delta(a_{k_\ell}, \hat{a}_{k_\ell}^{(x_{k_\ell})}) \cdot \prod_{r=D+1}^{N} \delta(a_r, \hat{a}_r^{(x_r)}) \tag{20}$$
$$= \sum_{\lambda_{k_1}, \ldots, \lambda_{k_n}} \Pr_{A_{j_1}^{j_m} A_{k_1}^{k_n} | X_{j_1}^{j_m} X_{k_1}^{k_n}}(a_{j_1}, \ldots, a_{j_m}, \lambda_{k_1}, \ldots, \lambda_{k_n} \mid 0, \ldots, 0, 0, \ldots, 0) \cdot \prod_{\ell=1}^{n} \delta(a_{k_\ell}, \hat{a}_{k_\ell}^{(x_{k_\ell})}) \cdot \prod_{r=D+1}^{N} \delta(a_r, \hat{a}_r^{(x_r)}) \tag{21}$$
$$= \Pr_{A_{j_1}^{j_m} | X_{j_1}^{j_m} X_{k_1}^{k_n}}(a_{j_1}, \ldots, a_{j_m} \mid 0, \ldots, 0, 0, \ldots, 0) \cdot \prod_{\ell=1}^{n} \delta(a_{k_\ell}, \hat{a}_{k_\ell}^{(x_{k_\ell})}) \cdot \prod_{r=D+1}^{N} \delta(a_r, \hat{a}_r^{(x_r)}) \tag{22}$$
$$= \Pr_{A_{j_1}^{j_m} | X_{j_1}^{j_m}}(a_{j_1}, \ldots, a_{j_m} \mid 0, \ldots, 0) \cdot \prod_{\ell=1}^{n} \delta(a_{k_\ell}, \hat{a}_{k_\ell}^{(x_{k_\ell})}) \cdot \prod_{r=D+1}^{N} \delta(a_r, \hat{a}_r^{(x_r)}) \tag{23}$$
$$= \Pr_{A_1 \ldots A_N | X_1 \ldots X_N}(a_1, \ldots, a_N \mid x_1, \ldots, x_N) \,, \tag{24}$$

where Eq. (19) follows directly from the definition of shared randomness; Eq. (20) uses the definition of the new strategy $\widehat{\Pr}$ described above; Eq. (21) follows from the observation that only terms satisfying $\lambda_{j_t} = a_{j_t}$ for $t \in \{1, \ldots, m\}$ have nonzero contribution; Eq. (22) is obtained by summing over all possible outputs of players $k_1, \ldots, k_n$; Eq. (23) follows from the nonsignalling condition applied to players $j_1, \ldots, j_m$; and finally, Eq. (24) is an immediate consequence of Eq. (17) being equal to Eq. (13).

Therefore, the new classical strategy reproduces exactly the output distribution of the initial strategy, which concludes the proof. □

References

1. D Mayers, A Yao, Self testing quantum apparatus. *Quantum Inf. Comput.* **4**, 273–286 (2004).
2. R Colbeck, A Kent, Private randomness expansion with untrusted devices. *J. Phys. A: Math. Theor.* **44**, 095305 (2011).
3. T Kraft, S Designolle, C Ritz, N Brunner, O Gühne, M Huber, Quantum entanglement in the triangle network. *Phys. Rev. A* **103**, L060401 (2021).
4. BS Tsirelson, Quantum generalizations of Bell's inequality. *Lett. Math. Phys.* **4**, 93–100 (1980).
5. X Coiteux-Roy, E Wolfe, MO Renou, No bipartite-nonlocal causal theory can explain Nature's correlations. *Phys. Rev. Lett.* **127**, 200401 (2021).
6. PK Aravind, Quantum mysteries revisited again. *Am. J. Phys.* **72**, 1303–1307 (2004).
